# Development of a Rapid Mass Spectrometric Determination of AMP and Cyclic AMP for PDE3 Activity Study: Application and Computational Analysis for Evaluating the Effect of a Novel 2-oxo-1,2-dihydropyridine-3-carbonitrile Derivative as PDE-3 Inhibitor

**DOI:** 10.3390/molecules25081817

**Published:** 2020-04-15

**Authors:** Ilaria Cicalini, Barbara De Filippis, Nicola Gambacorta, Antonio Di Michele, Silvia Valentinuzzi, Alessandra Ammazzalorso, Alice Della Valle, Rosa Amoroso, Orazio Nicolotti, Piero Del Boccio, Letizia Giampietro

**Affiliations:** 1 Centre of Advanced Studies and Technologies (CAST), University “G. d’Annunzio” of Chieti-Pescara, 66100 Chieti, Italy; 2 Department of Medicine and Aging Sciences, University “G. d’Annunzio” of Chieti-Pescara, 66100 Chieti, Italy; 3 Department of Pharmacy, University “G. d’Annunzio” of Chieti-Pescara, 66100 Chieti, Italy; 4 Department of Farmacia-Scienze del Farmaco, University “A. Moro” of Bari, 70126 Bari, Italy

**Keywords:** phosphodiesterases activity, AMP, cyclic AMP, tandem mass spectrometry, dihydropyridine, docking studies

## Abstract

A simple, quick, easy and cheap tandem mass spectrometry (MS/MS) method for the determination of adenosine monophosphate (AMP) and cyclic adenosine monophosphate (cAMP) has been newly developed. This novel MS/MS method was applied for the evaluation of the inhibitory effect of a novel 2-oxo-1,2-dihydropyridine-3-carbonitrile derivative, also named DF492, on PDE3 enzyme activity in comparison to its parent drug milrinone. Molecule DF492, with an IC_50_ of 409.5 nM, showed an inhibition of PDE3 greater than milrinone (IC_50_ = 703.1 nM). To explain the inhibitory potential of DF492, molecular docking studies toward the human PDE3A were carried out with the aim of predicting the binding mode of DF492. The presence of different bulkier decorating fragments in DF492 was pursued to shift affinity of this novel molecule toward PDE3A compared to milrinone in accordance with both the theoretical and experimental results. The described mass spectrometric approach could have a wider potential use in kinetic and biomedical studies and could be applied for the determination of other phosphodiesterase inhibitor molecules.

## 1. Introduction

3′,5′-cyclic adenosine monophosphate (cAMP) and 3′,5′-cyclic guanosine monophosphate (cGMP) are intracellular second messengers that play a key role in many physiological processes. cAMP is synthesized from adenosine triphosphate (ATP), by adenylate cyclase (AC) and cGMP is synthesized by guanylate cyclase (GC), and they are metabolized and degraded by cyclic nucleotide phosphodiesterases (PDEs) [1,2].

Since the 1970s, different isoforms of PDEs have been described in mammalians, and these are grouped into 11 families based on structural similarity (PDE1–PDE11). PDEs are inhibited by different drugs blocking one or more PDE subtypes. PDE3 is described as a phosphodiesterase isoform involved in cardiovascular disease. This isoform comprises two subfamilies, PDE3A and PDE3B, showing different subcellular and tissue distributions. PDE3A is highly expressed in the cardiovascular system and is a subtype more abundant in platelets [3], while PDE3B is mainly present in adipocytes [4].

The inhibition of PDE3 increases the intracellular levels of cAMP and consequently the force of heart contractions. Milrinone is a specific PDE3A inhibitor and is generally used to treat acute congestive heart failure, pulmonary hypertension or chronic heart failure [5]. This drug has cardioprotective effects associated with an increase of heart muscle contraction, vasodilatation and reduction of inflammatory damage produced by tumor necrosis factor-alpha (TNF-alpha) [6]. Milrinone has also been reported to reduce ischemia-reperfusion injury to the rat heart [7], lung [8] and liver [9].

Moreover, several studies demonstrated that milrinone has the capability to decrease apoptosis and release free oxygen radicals, with a consequent anti-inflammatory effect [10,11,12]. Vasodilatation, anti-inflammatory and antiaggregant effects of milrinone on regulating microcirculation cause an increase of tissue perfusion [13].

Likewise, milrinone increases calcium entry into cells by activating the calcium sensitive signaling cascades, resulting in a considerable neuroprotective effect on ischemic brain [14]. Milrinone has also emerged as an option to treat delayed cerebral ischemia after a subarachnoid hemorrhage [15]. During the last few years, milrinone has gained interest especially for an innovative use as intravenous salvage therapy in acute internal carotid artery use [16].

**Figure 1:**
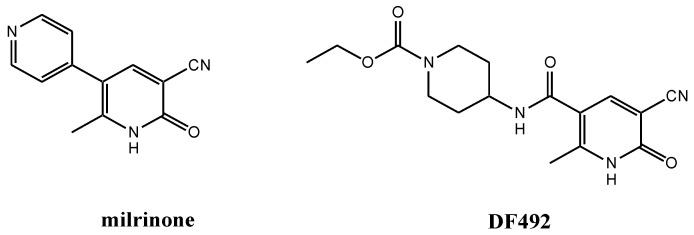
Chemical structures of milrinone and new analogue DF492.

In the past, several chemical modifications of milrinone have been made in order to synthesize new inotropic compounds with better cardiotonic activity and fewer side effects [17,18,19,20,21]. These chemical modifications were carried out following important information revealed about the crystal structure of PDE3B [22]. The interesting results obtained prompted us to investigate new milrinone analogues in order to enlarge our knowledge about the milrinone structure-activity relationship (SAR). For these reasons, we synthesized new compounds with modifications at the 3-position of milrinone, where the 4-pyridyl moiety was simply removed or replaced with an acid, ester or amide group [23]. All compounds were tested to evaluate the intracellular calcium increase in single livingH9C2 cardiac cells. Among them, only the amide derivative, DF492, (Figure 1) at a concentration of 10 µM in the presence of the KCl dependent [Ca^2+^]_i_ transient of cardiac myocytes, is able to extend it in a significant manner.

To date, the most applied methods for the indirect measurement of PDE3 activity regard the quantification of cAMP by using immunological assay. However, this kind of analytical technique may require an acetylation step and radiolabeling to improve the sensitivity [24]. Moreover, few high-performance liquid chromatography (HPLC) coupled to fluorescence or to photodiode methods are described. These latter methods allow good precision and accuracy but require high sample volumes and time demanding analyses [25,26]. Currently, tandem mass spectrometry (MS/MS) is becoming a valuable tool for the selective analysis of multiple biomarkers due to highly selective multiple reaction monitoring (MRM) in a simultaneous measurement, allowing small sample volumes and shorter analyses [27].

Table 1 summarizes the most applied methods for the determination of AMP and cAMP and/or PDE activity describing their advantages/disadvantages in term of costs, time consuming and performances.

Here, we developed and applied a simple and fast MS/MS method for the study of PDE3 enzyme activity by simultaneously measuring AMP and cAMP without chromatographic separation and avoiding internal standards use, making the method easy to use and inexpensive. Then, we applied the described method for the evaluation of the inhibitory effect of DF492 on PDE3 activity. Moreover, we reported a computational study to explain the interactions of DF492 at the binding site of PDE3A compared to milrinone, its parent drug.

## 2. Materials and Methods

### 2.1. Synthesis

Compound DF492 was synthesized according to previous procedures reported elsewhere [23].

### 2.2. Chemicals and Reagents

Working standards of AMP and cAMP were obtained from Sigma-Aldrich (St. Louis, MO, USA). The phosphodiesterase enzyme 3A (PDE3A) was purchased from Calbiochem (San Diego, CA, USA). Ammonium formate (NH_4_COOH) and magnesium chloride (MgCl_2_) were obtained from Sigma-Aldrich; all other chemicals were of Liquid Chromatography-Mass Spectrometry LC-MS grade (Sigma-Aldrich, St. Louis, MO, USA).

### 2.3. Tandem Mass Spectrometry Analysis

Sample injection was obtained by using an Liquid Chromatography (LC) system 2795 Separation Module (Waters Corp, Milford, MA, USA), and the chromatography was performed at room temperature using a GromSaphir 110 C_18_ column (3 μmCartrige 60 × 2 mm) connected to a guard column Security Guard Cartrige C_18_ (4 × 2.0 mm) through an isocratic elution for 5 min. The mobile phase consisted of 80% H_2_O, 20% acetonitrile (ACN) and 0.2% formic acid (FA); the flow rate was set at 0.2 mL/min, and the injection volume was 20 μL.

The LC system was coupled with a High Capacity Ion Trap (HCT) mass spectrometer (Bruker Daltonics GmbH, Bremen, Germany) through an Electrospray Ionization (ESI)source operating in positive mode. A 35 nA current was applied on the capillary, while the nebulizer pressure was set at 40 psi, dry gas was set at 9.0 L/min and dry temperature was set at 365 °C.

Peak detention was performed using MRM of the transitions of m/z 348.2 →136.1 for AMP and *m/z* 330.1 → 136.1 for cAMP, with a scan time of 0.2 s. The mass scan was set from 50 to 500 *m/z*, and the fragmentation was performed by using helium as collision gas.

The LC-MS/MS method was developed and optimized using a solution of AMP and cAMP 0.1 μmol/mL dissolved in H_2_O and ACN 50:50 and 0.2% AF.

### 2.4. Preparation of Stock Solutions and Calibrations Standards

The stock solutions of AMP and cAMP were prepared in 5 mM ammonium formate buffer (pH 7.5) and 100 μM of MgCl_2_ at a concentration of 20 μmol/mL and 10 μmol/mL respectively. A reproducibility test was performed by analysing six replicates of a solution containing cAMP at 3.5 pmol/μL, AMP at 0.25 pmol/μL, DMSO (1 μL) using 5 mM ammonium formate buffer (pH 7.5) and 100 μM of MgCl_2_ as solvent to achieve a final volume of 200 μL.

A correlation curve was performed using five solutions prepared at increasing concentrations, specifically 0.35, 0.87, 1.75, 2.6 and 3.5 pmol/μL for cAMP and 0.25, 0.32, 0.4, 0.7 and 0.9 pmol/μL for AMP (*n* = 4).

### 2.5. Study of PDE3A Activity and Effects of PDE3A Inhibitors

Enzyme activity was investigated by preparing an enzymatic reaction mixture containing 10 μL of PDE3A 0.15 nmol/mL, 1 μL of DMSO, 89 μL of 5mM ammonium formate buffer (pH 7.5) and 100 μM of MgCl_2_as already described [28]. Reaction was initiated by addition of the substrate molecule (cAMP) at 7.0 nmol/mL (100 μL) and incubated at 37 °C. The reactions were stopped by placing the solutions at 100 °C; then, the samples were centrifuged for 5 min at 9280 rcf and stored at −20 °C until further analysis.

The inhibitory action of milrinone and DF492 was investigated by preparing an enzymatic reaction mixture containing 10 μL of PDE3A 0.15nmol/mL,1 μL of inhibitors at increasing concentrations (20–1200 nM and 20–600 nM, respectively), 89 μL of 5 mM ammonium formate buffer (pH 7.5) and 100 μM of MgCl_2_. Reaction was initiated by addition of cAMP at 7.0 nmol/mL (100 μL) and incubated at 37 °C. The reactions were stopped, centrifuged and stored as previously reported.

### 2.6. Data Analysis

Mass spectrometry data obtained were processed using GraphPad Prism v. 5.02 software. The PDE3A activity was determined as a ratio of peak area of AMP (product) and the sum of peak areas of AMP and cAMP (substrates); data were expressed as mean ± standard deviation (SD). Inhibitory actions of DF492 and milrinone were investigated by performing a non-linear regression using a build-model called dose-response inhibition and by calculating IC_50_ for each inhibitor. Data were expressed as mean ± standard deviation (SD) versus logarithm of inhibitor concentration.

### 2.7. Docking Studies

Molecular docking studies toward the human PDE3A were carried out with the aim of predicting the binding mode of the molecule DF492 and to explain its inhibitory potential. As the crystal solved structure of PDE3A is not available in the Protein Data Bank (PDB), we employed a model recently created and validated by Muñoz-Gutiérrez et al. using homology modelling and molecular dynamics simulations [36]. This model was generated based on the X-ray structure of the catalytic domain of PDE3B (PDB entry: 1SO2) provided that an identity of 66% was found by considering the catalytic residues from 674 to 1140 of PDE3A vs PDE3B. It is noteworthy that no differences were observed for those residues with a clear role for binding interactions. This homology model was used as input for the protein preparation wizard, available from the Schrödinger suite [37]. Seven water molecules together with the two magnesium ions were kept because of their functional and catalytic functions. Particularly, six out of seven water molecules are crucial for the coordination of the two magnesium ions [36], while the other is involved in a relevant water bridge interaction within the PDE3A binding pocket. Next, the ligand structures to be docked were optimized using the LigPrep tool [38] allowing the generation of the possible ionization states at pH from 6 to 8 as well as all the generation of the possible tautomers. First, the energetic gridbox was centered on the center of mass of PZO14, the cognate ligand of PDE3B, which included a dihydropyridazinone ring very similar to the dihydropyridine ring of DF492 and milrinone, a well-known inhibitor of PDE3 whose X-ray structure is however still missing. The posing of PZO14 and its high similarity to DF492 and milrinone was used as criteria to drive and assess docking studies.

Glide standard precision (SP) was used for docking studies by implementing default settings. The molecular mechanics/generalized Born surface area (MM-GBSA) approach was also investigated in order to calculate the binding free energies (ΔG) between protein and ligands [39]. In the MM-GBSA method, the binding free energy (ΔG_bind_) between the ligand and the target complex is calculated as:

ΔG_bind_ = ΔE_MM_ + ΔG_pol_+ ΔG_np_

where ΔE_MM_ term includes bond stretching, angle bending, torsion rotation, van der Walls, and electrostatic contributions; ΔG_pol_ term represents the polar contribution to the solvation free energy, while ΔG_np_ term stands for the non-polar contribution. To carry out our analyses, we used the Prime package available in the Schrodinger software [38]. Satisfactorily, the top-scored pose of PZO14 carried out from re-docking analysis returned a Root Mean Square Deviation (RMSD) value equal to 0.40 Å and docking score and ΔG_bind_ values equal to −12.561 kcal/mol and −109.78 kcal/mol, respectively.

## 3. Results and Discussion

### 3.1. Tandem Mass Spectrometry Method Assessment

**Figure 2:**
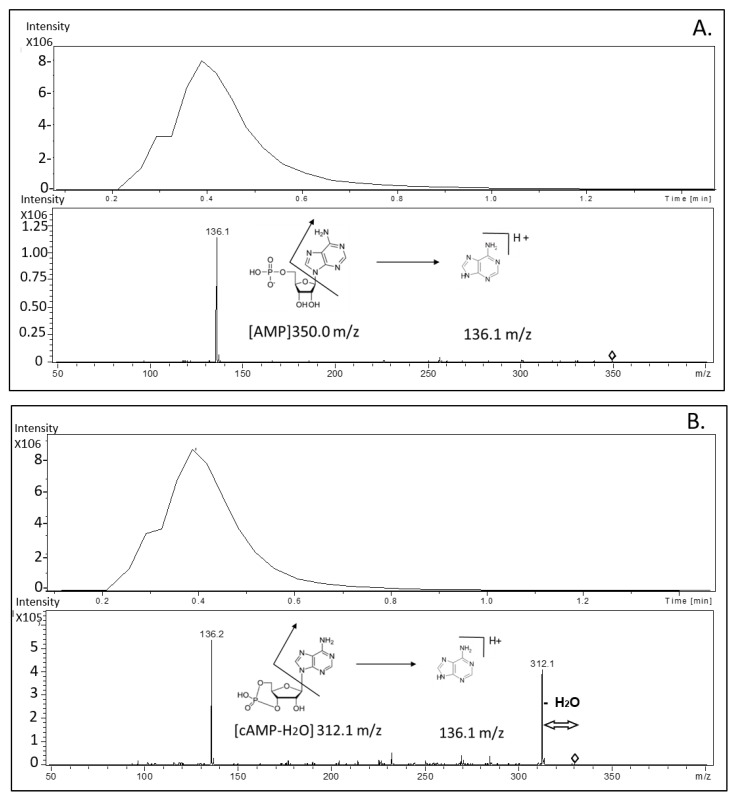
Representative chromatograms and mass spectrum obtained from tandem mass (MS/MS) analysis. Panel (**A**) and (**B**) show chromatogram peaks and mass spectrum from adenosine monophosphate (AMP) and cyclic adenosine monophosphate (cAMP) fragmentation, respectively.

To determine the amount of AMP and cAMP, two different mass transitions were used for each molecule, as shown in the representative chromatograms and mass spectrum obtained from MS/MS analysis in Figure 2. Panel A and B show a chromatogram peak and mass spectrum from AMP and cAMP fragmentation, respectively.

The proposed method did not provide a chromatographic retention for AMP and cAMP, since the column was exclusively used as an in-line filter to limit samples impurities.

Intra and inter-day reproducibility of the proposed method was performed comparing cAMP and AMP mean peak areas (coefficient of variation (CV%) < 13.1%), as described in Table 2.

**Figure 3:**
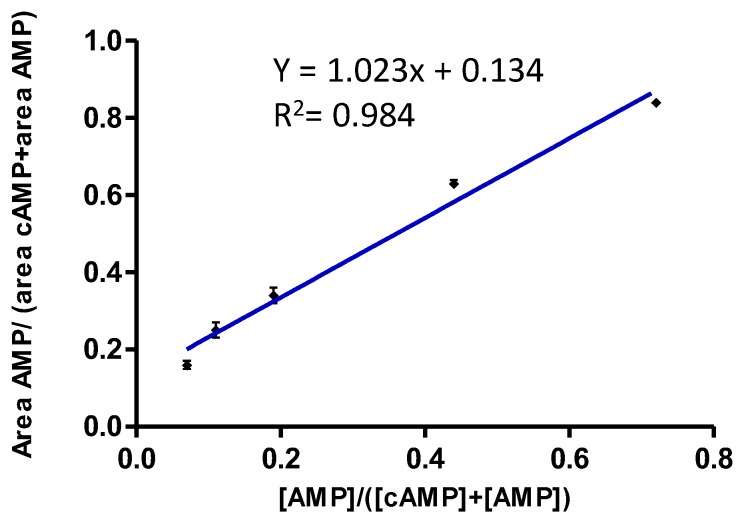
A correlation curve was performed using five solutions prepared at increasing concentrations, specifically 0.35, 0.87, 1.75, 2.6 and 3.5 pmol/μL for cAMP and 0.25, 0.32, 0.4, 0.7 and 0.9 pmol/μL for AMP (*n* = 4).

To test the linearity of the method we performed a correlation curve by estimating peak areas of AMP and cAMP; the data obtained were then correlated to their concentrations according to the following formulas: area AMP/(area AMP+ area cAMP) and [AMP]/([AMP] + [cAMP]). Five different solutions (A, B, C, D, E) with increasing concentrations of AMP and decreasing concentrations of cAMP were used, specifically 0.35, 0.87, 1.75, 2.6 and 3.5 pmol/μL for cAMP and 0.25, 0.32, 0.4, 0.7 and 0.9 pmol/μL for AMP (*n* = 4). As reported in Figure 3, the regression equation was y = 1.023x+ 0.134 (R^2^ = 0.984), indicating good and linear correlation and reproducibility (relative standard deviation (RSD%) < 7.55).

On the basis of these results, we carried out enzymatic activity studies using the area ratio of the mass signals, regardless of the absolute concentration of AMP and cAMP.

The developed method was applied to the study of PDE3A activity without inhibitors and subsequently with milrinone and DF492 as inhibitors.

**Figure 4:**
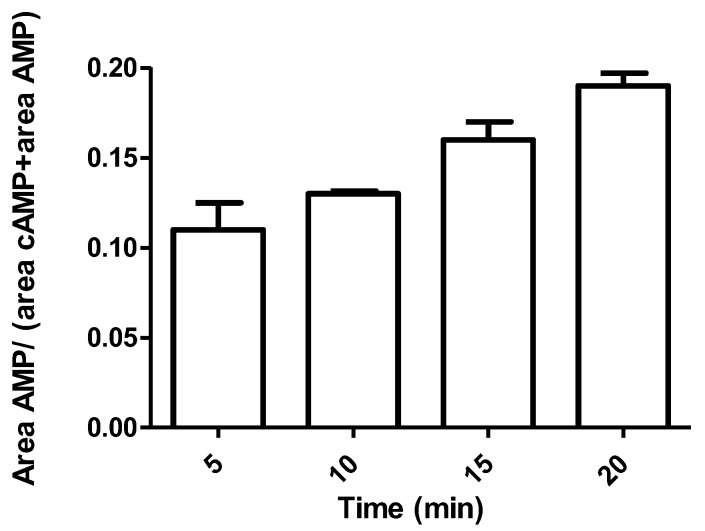
Enzymatic activity evaluated by the ratio of AMP area and AMP+cAMP areas after enzyme incubation at different time points (0, 5, 10, 15, 20 min after incubation) (*n* = 3).

PDE3A activity was investigated performing an experiment by using cAMP at 7 nmol/mL as substrate and PDE3A at 0.15 nmol/mL, as described in previous sections. Enzymatic activity was calculated at different time points: 0, 5, 10, 15 and 20 min after enzyme incubation. Through the MS/MS method developed, at each point, cAMP (substrate) and AMP (product) peak areas were detected, and the area ratio was calculated. The results are shown using a histogram (Figure 4). Data show that already after 10 min of incubation appreciable enzymatic activity is present and the RSD% observed has the lowest value (RSD% = 1.20). Thus, we decided to observe the effects of inhibitors on PDE3A activity after 10 min of incubation.

### 3.2. Enzymatic Activity With and Without Inhibitors 

Firstly, the effect of milrinone was investigated by performing an experiment at increasing concentrations of the inhibitor (20–1200 nM), and a percentage residual activity was estimated, as reported in Table 3. Three replicates were analyzed for each condition, and the average residual activity was calculated with the SD and relative SD% (RSD%).

Similarly, DF492 inhibitor effect was investigated by using an increasing concentrations of the inhibitor (20–600 nM), as reported in Table 4.

### 3.3. Comparison of the Inhibitory Effects of milrinone and DF492

**Figure 5:**
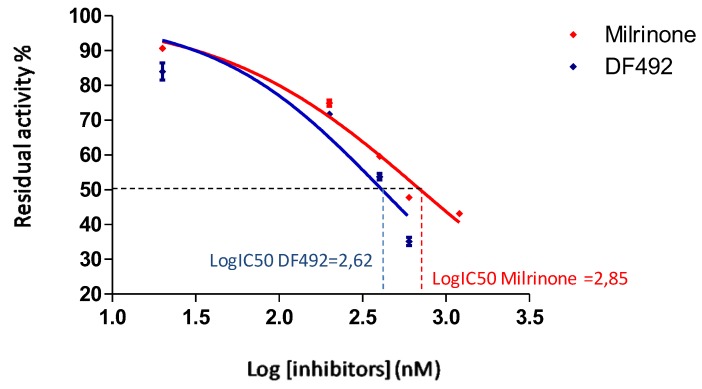
Non-linear regression evaluated by using a “dose-response inhibition” build-model to calculate the concentration of inhibitor that gives a response halfway between bottom and top (IC_50_). The figure shows the residual activity% versus the logarithm of milrinone (red line) and DF492 (blue line) concentrations and the calculated IC_50_.

As reported in Figure 5, we performed a non-linear regression using a “dose-response inhibition” build-model to calculate the concentration of inhibitor that gives a response halfway between bottom and top (IC_50_), using GraphPad Prism v 5.02 [40].

The graphs in Figure 5 show the trend of the percentage of residual enzyme activity as the inhibitor concentration increases, after carrying out a logarithmic transformation, performing a non-linear regression using a “dose-response inhibition” build-model, as previously described.

The red line identifies the trend of the milrinone, while the blue line indicates the trend of the DF492. As shown in Figure 5, the LogIC_50_ of milrinone was 2.85, while the LogIC_50_ of DF492 was 2.62.

Firstly, to choose the best model describing the inhibitory activity of milrinone and DF492, we performed a comparison in order to investigate which model fits best, for each data set (milrinone and DF492 activity). The considered models are “log inhibitors versus normalized response” and “log inhibitors versus normalized response-variable slope”. The first model assumes that the dose-response curve has a standard slope, called a Hill slope, of −1.0; the second one does not assume a standard slope but rather fits the Hill slope from the data, and this is called a variable slope model. In Table 5 we report the results obtained from the statistical comparison.

According to the results obtained for the milrinone data set, there is a significant difference between the two models (*p*-value = 0.0008) and the “log inhibitors versus normalized response-variable slope” is the preferred one. In contrast, for the DF492 data set there is not a significant difference between the two equations (*p*-value = 0.653). For this reason it was considered the “variable slope model” for each data set.

Such data processing highlights a significant difference between the two inhibitors considered: milrinone has an IC_50_ equal to 703.1 nM, while the IC_50_ of DF492 is 409.5 nM. These data suggest that DF492 possesses a better PDE3 inhibitory effect than milrinone. Probably, the replacement of the pyridine with a piperidine ring causes the capability of DF492 to better interact with PDE3.The larger groups of DF492, in place of the pyridine of the milrinone, probably increase its potency for better chances to engage extra binding site interactions. This observation is consistent with the fact that the ethyl 4-amido-1-piperidine carboxylate substituent could extend into a large hydrophobic pocket; this increase of the size of the group would optimize interactions with the protein [22].

### 3.4. Molecular Docking

**Figure 6:**
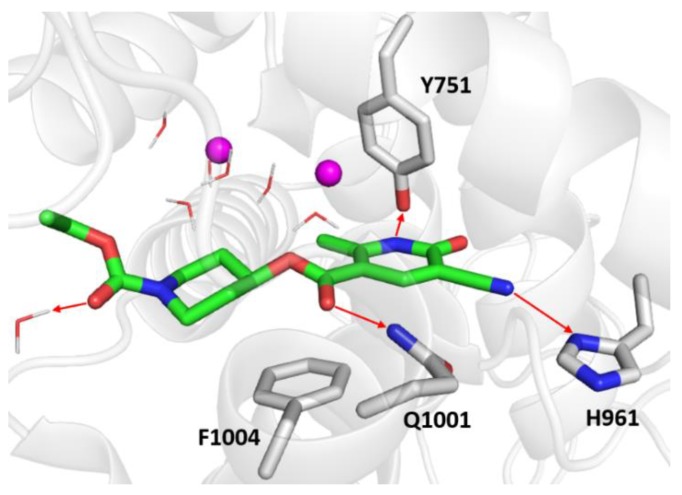
Zoomed in view of the PDE3A binding site. DF492is rendered as green sticks in the representation, the most relevant residues are reported as gray sticks, the magnesium ions are indicated as violet spheres, and the water molecules are depicted as wireframes. The red arrows indicate the hydrogen bonds.

To better understand the greater PDE3 inhibitory effect of DF492 compared to milrinone, a molecular docking study toward the human PDE3A was carried out with the aim of predicting the binding mode of DF492 and to explain its inhibitory potential. Figure 6 shows the binding mode of the top-scored solution of DF492 in a PDE3A binding pocket.

**Figure 7:**
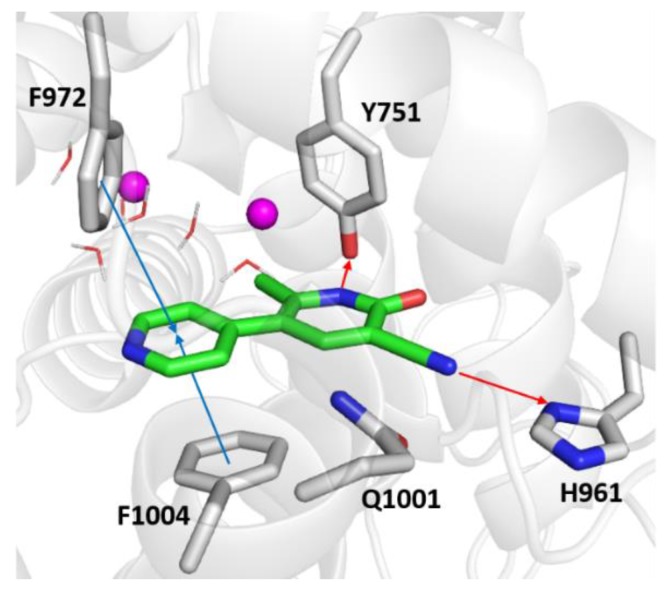
Zoomed in view of the PDE3A binding site. Milrinone is rendered as green sticks in the representation, the most relevant residues are reported as gray sticks, the magnesium ions are indicated as violet spheres, and the water molecules are depicted as wireframes. The red and blue arrows indicate the hydrogen bonds and the π-π interactions.

The docking score and ∆G_bind_ values were equal to −9.007 kcal/mol and −50.55 kcal/mol, respectively. As far as DF492 (Figure 6) is concerned, the nitrogen atom of the dihydropyridine ring engages a hydrogen bond with the hydroxyl group of Tyr751, the cyano group makes a hydrogen bond with side chain of His961, and two carbonyl groups are involved in hydrogen bonds with the side chain of Gln1001 and with a functional water molecule of the binding pocket. Furthermore, a sandwich-like conformation between Phe1004 and the dihydropyrazine ring can be observed, which is very similar to the π-stacking interaction occurring between Phe1004 and the pyridine ring of milrinone (Figure 7).

As shown in Figure 7, the top-scored docking pose of milrinone was provided with a docking score and ΔG_bind_ value equal to −7.588 kcal/mol and to −25.79 kcal/mol, respectively. Likewise, with DF492, there are two hydrogen bonds engaged by the tri-substituted dihydropyridine ring and the side chains of Tyr571 and His961. In addition, π-π interactions occurred between its pyridine ring with Phe1004 and Phe972. Milrinone can experience hydrophobic interactions with Tyr751, His752, His756, Asp950, Ile951, Asn952, Gly953, Pro954, Lys956, Leu962, Trp964, Thr965, Ile968, Val969, Phe972, Phe989, Met990, Leu1000, Gln1001, Ser1003, Phe1004, Ile1005, Ile1008 and Val1009, most of these also being visited by DF492.

The herein reported computational studies shed light on the interactions of DF492 at the binding site of PDE3A. Importantly, the design of DF492 was inspired by milrinone whose tri-substituted dihydropyridine ring was kept unchanged for anchoring at the binding site. On the other side, the inclusion of different bulkier decorating fragments was pursued to shift affinity of DF492 toward PDE3A compared to milrinone according to both the theoretical and experimental results [41].

## 4. Conclusions

Cyclic nucleotides are intracellular second messengers playing a key role in many physiological processes, in various cell types and tissues. To turn off their signalling, cAMP and cGMP are degradated by PDEs, in particular PDE3is an important regulator of cAMP-mediated responses within the cardiovascular system, with distinct cellular and subcellular locations. Indeed, PDE inhibitors, such as milrinone, have aroused much interest as a group of potential anti-inflammatory and anti-remodeling drugs, since the cardioprotective and neuroprotective effects of the consequent increase of cAMP are well known [6,14].

Here, we described an MS/MS method for a fast, cheap, specific and reproducible determination of phosphodiesterase activity through the monitoring of AMP and cAMP levels during catalytic reaction. The developed method was applied to compare two PDE3 inhibitor molecules, milrinone and DF492, with the latter showing a greater inhibitory power. This rapid method is extremely simple and reproducible and could be useful for exploratory and preliminary studies aimed at developing deeper tests. In addition, unlike most MS/MS applications, our method does not require isotopic molecules as internal standards, since the quantification of PDE3 activity includes an internal normalization. This method could be applied for PDE activity measurements from biological fluids such as CSF and plasma, as well as from cellular systems, for example homogenized tissue, as reported in literature [27,42]. Moreover, docking studies showing interactions of DF492 at the binding site of PDE3A can explain at a molecular level the observed better inhibitory effect with respect to milrinone.

In summary, we have described a MS/MS approach that could have a wider potential use in kinetic and biomedical studies. Furthermore, the high selectivity of the method could be applied to the study of different activities of phosphodiesterase, potentially being used for the determination of other phosphodiesterase inhibitor molecules. Moreover, the computational studies confirmed the shift affinity of DF492 toward PDE3A compared to milrinone, in agreement with experimental results.

## Figures and Tables

**Table 1:** Advantages and disadvantages of methods used for the quantification of adenosine monophosphate (AMP) and cyclic adenosine monophosphate (cAMP) or for the measurement of phosphodiesterases (PDE) activity.

Method	AMP	cAMP	PDE Enzyme Activity	Advantages	Disadvantages	Ref.
**HPLC-MS/MS**	+	+		HPLC is associated with speed, efficiency and accuracy; it can be automated and requires minimal training	can be costly, requires a large amount of expensive organics, has low sensitivity for certain compounds	[28]
**CD Spectroscopy**			+	pre-steady and steady-state kinetics; real-time detection	micromolar detection limit	[29]
**qPCR**			+	dynamic range of quantification, high sensitivity and precision	not ideal for multiplexing, setting up requires high technical skills, high equipment cost, intra- and inter-assay variation, RNA lability, DNA contamination	[30]
**IHC**			+	*Fluorescent detection:* multiplexing, good target co-localization, high dynamic range *Chromogenic detection:* sensitivity and long-lasting signal	*Fluorescent detection:* low sensitivity, susceptible to photobleaching *Chromogenic detection*: narrow dynamic range	[30]
**ELISA immunoassays**	+	+		accuracy, high sensitivity, specificity	laborious assay procedure	[31]
**RIA**		+		implicity and high sensitivity	a scintillation counter is required	[32]
**AlphaScreen assay**		+		high sensitivity, rapid and simple, great dynamic range	requires special instrument capable to read A-counts	[33]
**Transcreener assay**	+			a single set of detection reagents can be used for all of the enzymes in a family and all acceptors substrates	no need for fluorescently labelled substrates, no signal background, expensive kit	[34]
**IMAP-FP**			+	an antibody-independent system	may interfere with other negatively charged reactants	[35]
**IMAP-TR-FRET**			+	ease of detection for kinase inhibitors	may interfere with ATP and other negatively charged compounds in high concentrations	[35]

HPLC-MS: high performance liquid chromatography-mass spectrometry; CD: circular dichroism; IHC: immunohistochemistry; qPCR: quantitative polymerase chain reaction; ELISA: enzyme-linked immunosorbent assay; RIA: radioimmunoassay; IMAP/FP: IMAP (Immobilized Metal Ion Affinity Particle) assay in fluorescence polarization detection mode; IMAP/TR-FRET: IMAP assay/time-resolved fluorescence energy transfer.

**Table 2:** Intra- and inter-day assay of cAMP and AMP by comparing mean peak area, in terms of standard deviation (SD) and coefficient of variation CV%.

Analyte	Intra-day Assay			Inter-day Assay		
	Mean Area (*n* = 8)	SD (*n* = 8)	CV%	Mean Area (*n* = 6)	SD (*n* = 6)	CV%
**cAMP** **(1.75 pmol/** **µ** **l)**	410,008.4	36,306.9	8.85	358,897.8	25,369.85	7.06
**AMP** **(0.25 pmol/** **µ** **l)**	137,478.4	17,339.6	12.6	120,487.7	15,879.38	13.17

**Table 3:** Residual enzymatic activity % calculated by adding different solutions at increasing concentrations of milrinone, as inhibitor. SD: Standard Deviation; RSD: Relative Standard Deviation.

[milrinone] (nM)	Residual *Activity* %	SD (n = 3)	RSD%
20	90.73	1.03	1.13
200	74.95	1.53	2.04
400	59.62	0.14	0.23
600	47.83	0.84	1.75
1200	43.18	0.97	2.24

**Table 4:** Residual enzymatic activity % calculated by adding different solutions at increasing concentrations of DF492, as inhibitor.

[DF492] (nM)	Residual *Activity* %	SD (n = 3)	RSD%
20	84.04	4.25	5.05
200	71.75	0.69	0.96
400	53.77	1.65	3.06
600	35.18	2.1	5.97

**Table 5:** Statistical comparison between two models considered: “log inhibitors versus normalized response” and “log inhibitors versus normalized response − variable slope” for each inhibitor. The table lists the LogIC_50_ and IC_50_ obtained from the comparison, the Hill slope considered and the preferred model resulted. ** means *p*-value < 0.01, NS means Not significant at t test.

	Milrinone		DF492	
	log(inhibitor) vs. Normalized Response	log(inhibitor) vs. Normalized Response − Variable slope	log(inhibitor) vs. Normalized Response	log(inhibitor) vs. Normalized Response − Variable slope
**LogIC_50_**	2.808	2.847	2.612	2.617
**IC_50_**	643.2	703.1	409.5	414.1
**Hill slope**	−1.0	−0.7113	−1.0	−0.8530
**P value**	0.0008 (**)		0.6527 (NS)	
**Preferred model**	log(inhibitor) vs. normalized response − Variable slope		---	
